# Ethanol-Mediated Regulation of Cytochrome P450 2A6 Expression in Monocytes: Role of Oxidative Stress-Mediated PKC/MEK/Nrf2 Pathway

**DOI:** 10.1371/journal.pone.0035505

**Published:** 2012-04-18

**Authors:** Mengyao Jin, Anil Kumar, Santosh Kumar

**Affiliations:** Division of Pharmacology and Toxicology, School of Pharmacy, University of Missouri-Kansas City, Kansas City, Missouri, United States of America; The University of Kansas Medical Center, United States of America

## Abstract

Cytochrome P450 2A6 (CYP2A6) is known to metabolize nicotine, the major constituent of tobacco, leading to the production of toxic metabolites and induction of oxidative stress that result in liver damage and lung cancer. Recently, we have shown that CYP2A6 is induced by ethanol and metabolizes nicotine into cotinine and other metabolites leading to generation of reactive oxygen species (ROS) in U937 monocytes. However, the mechanism by which CYP2A6 is induced by ethanol is unknown. In this study, we have examined the role of the PKC/Nrf2 pathway (protein kinase C-mediated phosphorylation and translocation of nuclear erythroid 2-related factor 2 to the nucleus) in ethanol-mediated CYP2A6 induction. Our results showed that 100 mM ethanol significantly induced CYP2A6 mRNA and protein (∼150%) and increased ROS formation, and induction of gene expression and ROS were both completely blocked by treatment with either a CYP2E1 inhibitor (diallyl sulfide) or an antioxidant (vitamin C). The results suggest the role of oxidative stress in the regulation of CYP2A6 expression. Subsequently, we investigated the role of Nrf2 pathway in oxidative stress-mediated regulation of CYP2A6 expression in U937 monocytes. Our results showed that butylated hydroxyanisole, a stabilizer of nuclear Nrf2, increased CYP2A6 levels >200%. Staurosporine, an inhibitor of PKC, completely abolished ethanol-induced CYP2A6 expression. Furthermore, our results showed that a specific inhibitor of mitogen-activated protein kinase kinase (MEK) (U0126) completely abolished ethanol-mediated CYP2A6 induction and Nrf2 translocation. Overall, these results suggest that CYP2E1-mediated oxidative stress produced as a result of ethanol metabolism translocates Nrf2 into the nucleus through PKC/MEK pathway, resulting in the induction of CYP2A6 in monocytes. An increased level of CYP2A6 in monocytes is expected to further increase oxidative stress in smokers through CYP2A6-mediated nicotine metabolism. Thus, this study has clinical relevance because of the high incidence of alcohol use among smokers, especially in HIV-infected individuals.

## Introduction

Cytochrome P450 (CYP) comprises a superfamily of heme proteins, which are most abundant in the liver and are involved in the metabolism of numerous xenobiotics, including the majority of therapeutic drugs [1]. To a lesser extent, they are also found in other organs, such as lung, brain, and kidney [2]–[4]. The CYP2A6 isozyme is known to metabolize nicotine, the major constituent of tobacco, causing tobacco-associated toxicities in both the liver and lung [5]. In addition, CYP2A6 activates multiple tobacco procarcinogens including 4-(methylnitrosamino)-1-(3-pyridyl)-1-butanone (NNK), leading to liver damage and lung cancer [5]. CYP2A6 is involved in approximately 3% of all CYP-mediated metabolisms of therapeutic drugs, such as pilocarpine, cyclophosphamide and tegafur. Furthermore, CYP2A6 has been implicated in drug-drug interactions in normal, as well as in polymorphic populations [6], [7].

In general, four major nuclear receptors are known to regulate different CYP isozymes [8]. These are: 1) Aryl hydrocarbon receptor (AhR), which is associated with the regulation of CYP1A1, 1A2, and 1B1; 2) Constitutive androstane receptor , which mediates the regulation of CYP2B enzymes; 3) Peroxisome proliferator-activated receptor (PPAR), which is associated with CYP2C expression; and 4) Pregnane X-receptor (PXR), which is important in regulating CYP2B and CYP3A enzymes. Although the pathway by which many CYP enzymes are regulated is known, the mechanism of CYP2A6 regulation is not clear. A previous study has shown that in the presence of PPARγ, the heterodimer PXR/retinoic X receptor is involved in the regulation of CYP2A6 in hepatocytes [9].

A recent study has shown that mouse CYP2A5 (analogous to human CYP2A6) is induced through oxidative stress resulting from ethanol consumption and nicotine treatment [10], [11]. Consistent with this, our previous study has shown that CYP2A6 is the most abundant CYP in U937 cells and is induced by ethanol [12], [13]. However, the mechanism of CYP2A6 induction by ethanol in monocytes is not known. Monocytes/macrophages are one of the major targets of HIV-1 infection and are also one of the major reservoirs for virus replication [14]. U937 is a human monocytic cell line and is utilized for HIV-1 related research [15]. The prevalence of smoking is 3 times higher in alcohol users compared to the normal population, and the prevalence of co-abuse is approximately 90% among HIV-1 infected individuals [16], [17]. Thus, it is important to determine the mechanism(s) responsible for alcohol-mediated induction of CYP2A6 in monocytes/macrophages. In the current study, we investigate the pathway by which ethanol induces CYP2A6 in the U937 monocyte cell line.

## Materials and Methods

### Materials

The U937 monocytic cell line was obtained from ATCC (Manassas, VA). Protease inhibitor cocktail, vitamin C, butylated hydroxyanisole (BHA), staurosporine, U0126, and SB600125 were bought from Sigma-Aldrich (St. Louis, MO). Diallyl sulfide (DAS) was bought from Alfa Aesar, Heysham (Lancs, UK). Roswell Park Memorial Institute (RPMI) 1640 and Dulbecco's modified eagle medium (DMEM) media were purchased from Mediatech Inc. (Manassas, VA). The Qiagen RNeasy kit was obtained from Qiagen, (Valencia, CA). Gene expression kits and primer probes (CYP2A6, Hs0071162_m1) were obtained from Life Technologies (Foster City, CA). Radioimmunoprecipitation assay (RIPA) buffer was obtained from Boston BioProducts, (Ashland, MA). The bicinchoninic acid (BCA) protein assay kit was obtained from Thermo Scientific (Rockford, IL). All primary and secondary antibodies were from Santa Cruz Biotechnology Inc. (Santa Cruz, CA). Luminata™ Crescendo western HRP substrate was obtained from EMD Millipore Corporation (Billerica, MA). NE-PER Nuclear and Cytoplasmic Extraction Reagents were purchased from Thermo Scientific (Rockford, IL). Dichlorofluoroscein diacetate (DCFDA) was purchased from Life Technologies (Grand Island, NY).

### Cell culture and treatments

U937 cells were grown in RPMI 1640 media with 1% gentamicin at 37°C in a humidified incubator containing 5% CO_2_. Ethanol treatment of U937 monocytes was performed at 100 mM as previously described [13]. Treatments with vitamin C, BHA, staurosporine, SB600125, and U0126 were initiated 1 h prior to ethanol treatment. However, DAS was pretreated for 15 min, prior to ethanol treatment according to the previous protocol [18]. For control samples, only media or appropriate solvents for each treatment group (alcohol with and without DAS, BHA, vitamin C, SB600125 and U0126) were used at each time point.

### RNA extraction and quantitative reverse transcriptase-polymerase chain reaction (qRT-PCR)

Total RNA was extracted using a Qiagen RNeasy kit based on manufacturer's protocols. For each reaction, RNA (100 ng) from the samples was reverse-transcribed into cDNA using the High-capacity cDNA Reverse Transcription Kit. qRT-PCR was performed using cDNA generated from the reverse transcription of RNA according to the supplier's instructions and the products were analyzed on the iCycler iQ system (BioRad Laboratories, Hercules, CA). Relative gene expression was calculated using β-actin (ACTB) as an endogenous control. Ethanol treatment did not alter ACTB gene expression significantly in our experimental conditions in U937 monocytes (data not shown).

### Western blotting

Total cell lysate was prepared using RIPA buffer, including 4% of 1× protease inhibitor cocktail. The protein concentrations were measured using the BCA protein assay kit. Western blotting was performed essentially as previously described [13]. Briefly, 20 µg of total proteins were run on a SDS-PAGE and then transferred to polyvinylidene fluoride membranes. Transferred blots were blocked in 5% nonfat dry milk followed by overnight incubation with primary antibody (1∶1000 dilution) and 2 h incubation with an appropriate secondary antibody (1∶1500 dilution). Proteins were detected using Luminata™ Crescendo western HRP substrate, and quantified using the Alpha Innotech FluorChem HD2 gel documentation system (Proteinsimple, Santa Clara, CA). The densitometry data were analyzed using AlphaEase FC StandAlone software (version 6.0.0.14; Alpha Innotech). β-tubulin served as internal loading control to normalize the expression of CYP2A6 from whole cell extract, while lamin B was used as an internal control for the nuclear extract.

### Nuclear fractions preparation

Nuclear and cytosolic fractions were separated using NE-PER Nuclear and Cytoplasmic Extraction Reagents, according to the manufacturer's protocol. Briefly, the cell pellet was resuspended in CER I buffer and incubated on ice for 10 min followed by centrifugation at 16,000×g at 4°C for 5 min. The supernatant (cytosolic fraction) was collected and stored at −80°C. The pellets were resuspended in NER buffer, followed by 20 min incubation on ice. Cell debris was removed by centrifuging at 16,000×g for 10 min and the nuclear fraction in the supernatant was stored at −80°C. Nuclear fractions were used to measure Nrf2 protein levels.

### ROS measurement by flow cytometry

The production of ROS was measured by flow cytometry using DCFDA as described previously [12], [13]. Briefly, the monocytes were treated with alcohol, either with or without inhibitors, using serum-free medium at different times in a 6-well plate followed by addition of 10 µM DCFDA. Cells were then harvested and resuspended in 1 ml PBS to measure the DCF emission at 525±20 nm using a flow cytometer (BD Biosciences, San Jose, CA). Mean fluorescence intensity (MFI) was measured and analyzed.

### Statistical analysis

Statistical analysis for qRT-PCR, western blotting, and ROS measurement data was performed to determine mean ± SD and p values using one-way ANOVA. A p value of ≤0.05 was considered significant.

## Results

### CYP2A6 is induced by CYP2E1-mediated ethanol metabolism

**Figure 1:**
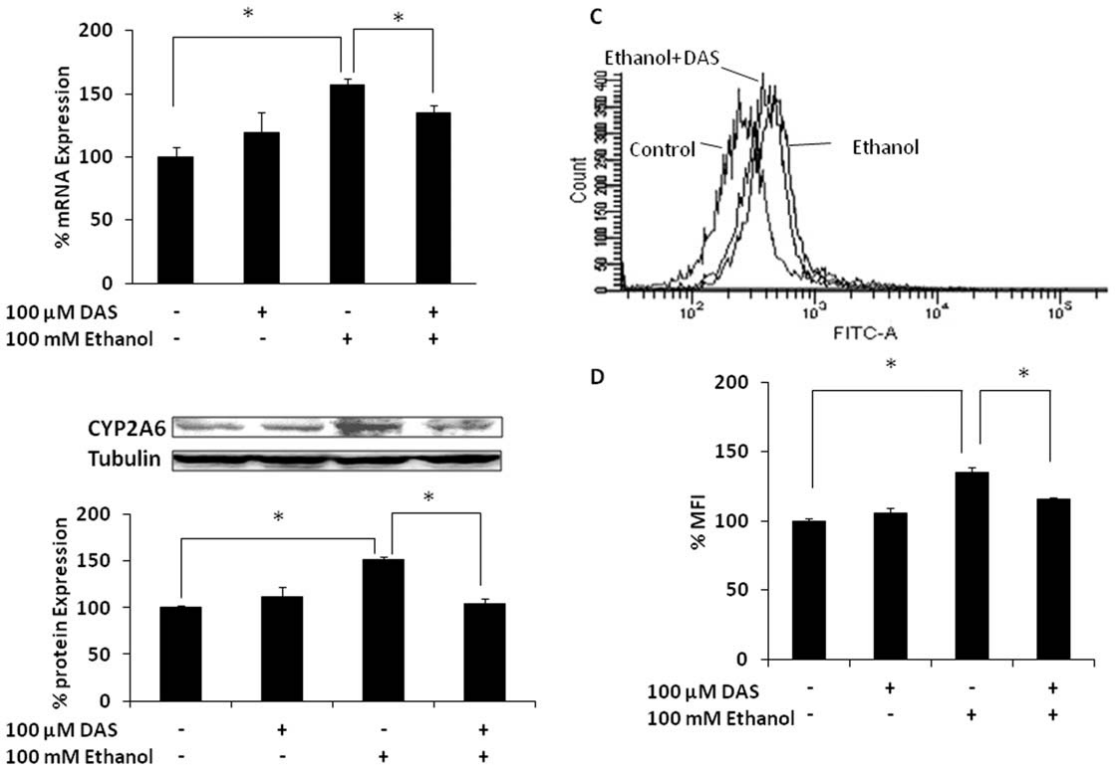
Effect of CYP2E1 selective inhibitor, DAS on ethanol-induced CYP2A6 expression level and oxidative stress. (A) CYP2A6 mRNA. (B) CYP2A6 protein. Both mRNA and protein expression levels were determined at 12 h in the presence of 100 mM ethanol (−/+100 µM DAS, 15 min prior to ethanol treatment). For each experiment, the mRNA/protein levels of various treatments were normalized relative to the untreated control, which has been set to 100%. Blots are representative of at least three replicates. (C) Representative figure of the determination of ROS levels at 2 h ethanol treatment with 0 and 100 µM DAS pretreatment for 15 min. The events (cell population) are presented in Y-axis and relative fluorescence intensity is presented in X-axis. (D) Bar graphs of MFI in the presence and absence of ethanol and DAS. The mean ± SD was calculated from at least triplicates and significance (p≤0.05; *) was determined using one-way ANOVA. The experiment was repeated twice.

U937 monocytes were pretreated in the absence or presence of 100 µM DAS to selectively inhibit CYP2E1 [19], followed by ethanol treatment for 12 h. Consistent with our previous study [13], 100 mM ethanol increased CYP2A6 mRNA and protein expression levels to 170% and 150% relative to control, respectively (Fig. 1A and B). As expected, DAS significantly inhibited ethanol-mediated CYP2A6 induction at both mRNA and protein levels. Similarly, consistent with our earlier finding [13], ethanol increased ROS formation by 25–30%, and DAS attenuated ethanol-induced ROS formation (Fig. 1C and D). DAS alone did not alter CYP2A6 expression or ROS production. In order to confirm that CYP2E1 is mainly involved in ethanol metabolism in U937 monocytes, we measured alcohol dehydrogenase (ADH) in monocytes. However, the level of ADH mRNA in U937 monocytes was undetectable (data not shown). Taken together, these results suggest that ethanol-induced CYP2A6 expression is mediated by the oxidative stress resulting from CYP2E1-dependent ethanol metabolism.

### Ethanol-mediated CYP2A6 induction is attenuated by vitamin C

**Figure 2:**
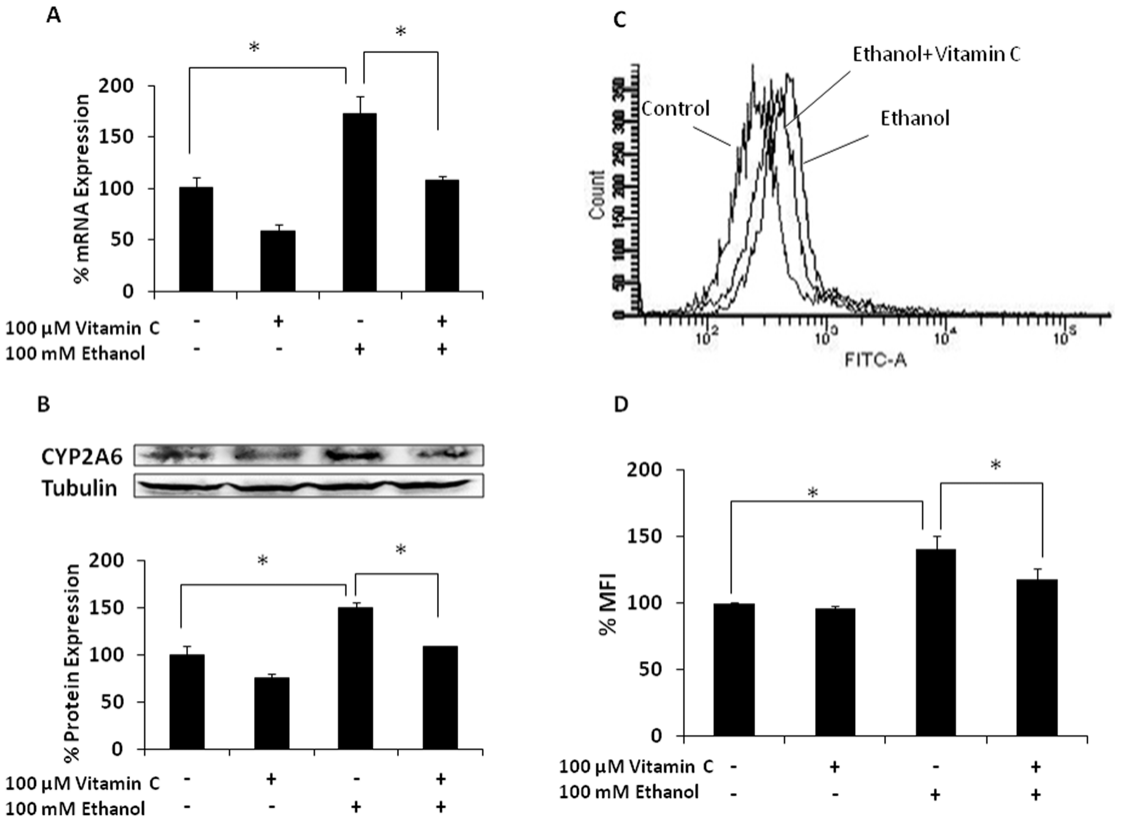
Effect of vitamin C on ethanol-induced CYP2A6 expression level and CYP2E1-mediated oxidative stress. (A) CYP2A6 mRNA. (B) CYP2A6 protein. Both mRNA and protein expression levels were determined at 12 h in the presence of 100 mM ethanol treatment (−/+100 µM vitamin C 1 h prior to ethanol treatment). Blots are representative of at least three replicates. For each experiment, the mRNA/protein levels of various treatments were normalized relative to the untreated control, which has been set to 100%. (C) Representative figure of the determination of ROS levels at 2 h ethanol treatment with 0 and 100 µM vitamin C pretreatment for 1 h. The events (cell population) are presented in Y-axis and relative fluorescence intensity is presented in X-axis. (D) Bar graphs of MFI in the presence and absence of ethanol and vitamin C. The mean ± SD was calculated from at least triplicates and significance (p≤0.05; *) was determined using one-way ANOVA. The experiment was repeated three times.

To examine the role of ethanol-metabolism induced oxidative stress in CYP2A6 expression, we treated the cells with 100 µM antioxidant vitamin C. Vitamin C significantly inhibited induction of CYP2A6 mRNA and protein by ethanol (Fig. 2A and B). As expected, vitamin C also attenuated ethanol-induced oxidative stress (Fig. 2C and D). These results suggested that ethanol-induced CYP2A6 expression is mediated through oxidative stress.

### Ethanol-induced CYP2A6 expression is mediated through nuclear translocation of Nrf2

**Figure 3:**
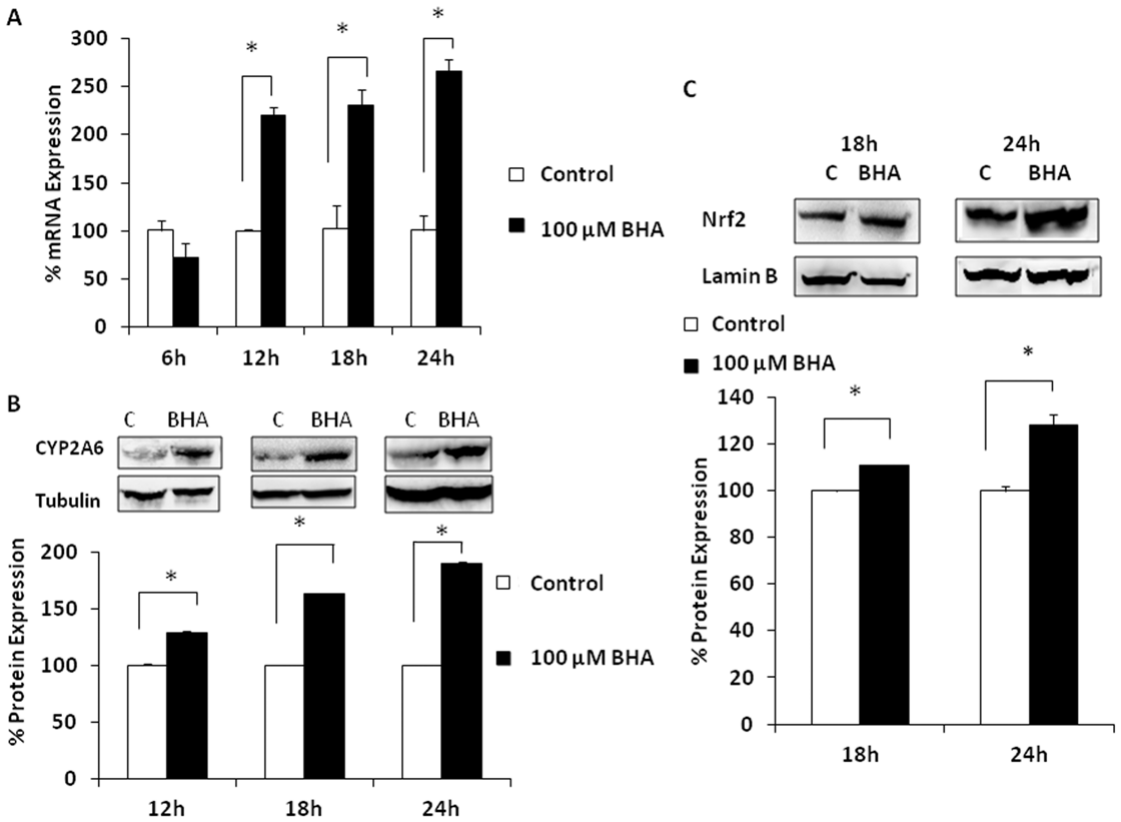
Effect of 100 µM BHA on CYP2A6 and Nrf2 expression levels. (A) CYP2A6 mRNA. (B) CYP2A6 protein. (C) Nrf2 protein from nuclear extracts. For each experiment, the mRNA/protein levels of various treatments were normalized relative to the untreated control, which has been set to 100%. Blots are representative of at least three replicates. The mean ± SD was calculated from at least triplicates and significance (p≤0.05; *) was determined using one-way ANOVA. The experiment was repeated twice.

Since oxidative stress-mediated translocation of nuclear erythroid 2-related factor 2 (Nrf2) is known to induce antioxidant genes, we tested whether this pathway is also involved in the regulation of the CYP2A6 enzyme. Treatment with 100 µM BHA, a stabilizer of Nrf2, enhanced CYP2A6 mRNA and protein levels by >200% and >150%, respectively (Fig. 3A and B). As expected, BHA also increased the level of Nrf2 in the nucleus at 12 and18 h (Fig. 3C). The BHA treatment at >24 h led to increased cell death (data not shown), which is consistent with a previous study that 100 µM BHA potentiates apoptosis after 24 h [20].

**Figure 4:**
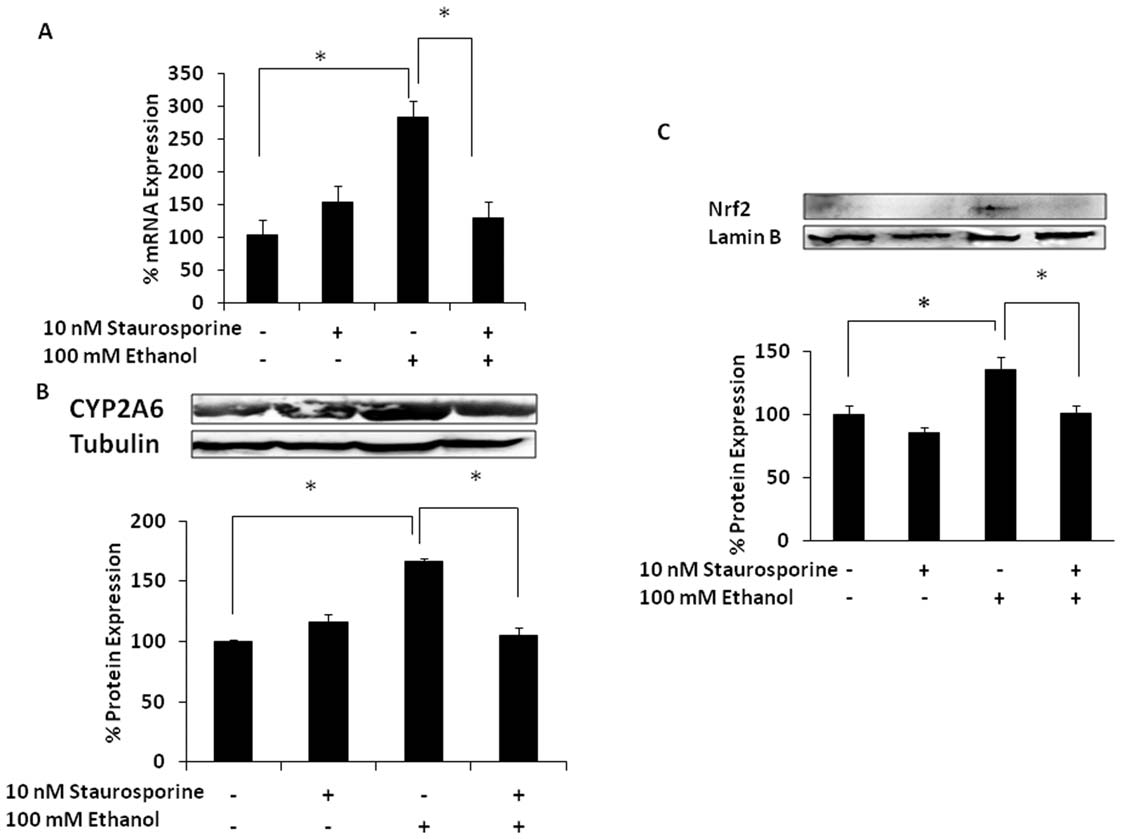
Effect of 10 nM staurosporine on ethanol-induced CYP2A6 and Nrf2 expression levels. (A) CYP2A6 mRNA. (B) CYP2A6 protein. (C) Nrf2 protein from nuclear extracts. Both mRNA and protein expression levels were evaluated at 12 h in the presence of 100 mM ethanol (−/+10 nM staurosporine 1 h prior to ethanol treatment). Blots are representative of at least three replicates. For each experiment, the mRNA/protein levels of various treatments were normalized relative to the untreated control, which has been set to 100%. The mean ± SD was calculated from at least triplicates and significance (p≤0.05; *) was determined using one-way ANOVA. The experiment was repeated three times.

To further examine the role of Nrf2 in CYP2A6 induction, U937 monocytes were pretreated with staurosporine, an inhibitor of PKC that also inhibits translocation of Nrf2 into the nucleus. Staurosporine abolished ethanol-mediated CYP2A6 mRNA and protein induction (Fig. 4A and B). As expected, staurosporine also decreased ethanol-induced Nrf2 expression in the nucleus (Fig. 4C). Overall, these results suggest that CYP2A6 induction is associated with increased translocation of Nrf2 into the nucleus.

### Ethanol induces CYP2A6 by the activation of the MEK pathway

**Figure 5:**
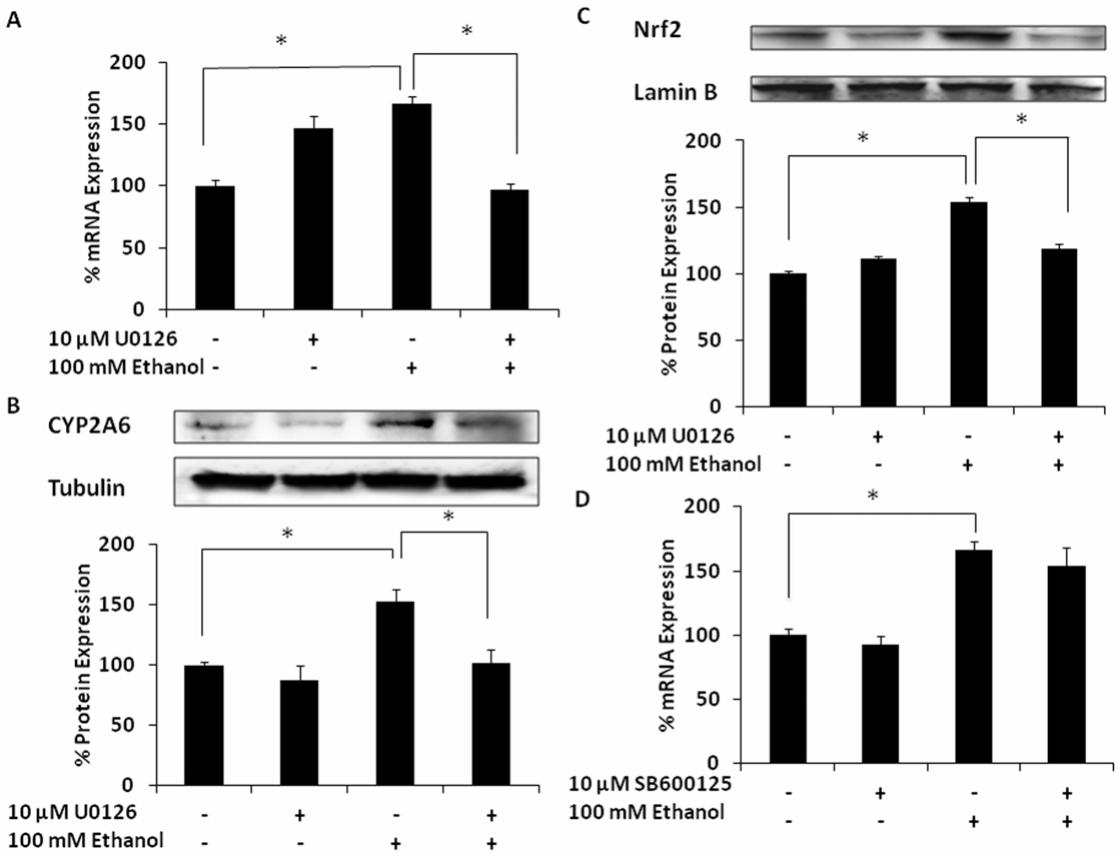
Effect of 10 µM U0126 (MEK inhibitor), as well as 10 µM SB600125 (JNK inhibitor), on ethanol-induced CYP2A6 and Nrf2 expression levels. (A) CYP2A6 mRNA. (B) CYP2A6 protein. (C) Nrf2 protein from nuclear extracts. Both mRNA and protein expression levels were evaluated at 12 h in the presence of 100 mM ethanol (−/+10 µM U0126 or SB600125 1 h prior to ethanol treatment). For each experiment, the mRNA/protein levels of various treatments were normalized relative to the untreated control, which has been set to 100%. Blots are representative of at least three replicates. The mean ± SD was calculated from at least triplicates and significance (p≤0.05; *) was determined using one-way ANOVA. The experiment was repeated twice.

To further investigate the signaling pathway(s) involved in ethanol-mediated Nrf2 translocation into the nucleus and CYP2A6 induction, we pretreated U937 monocytes using multiple inhibitors of the oxidative stress-PKC mediated pathway. The inhibitor of mitogen-activated protein kinase kinase (MEK), U0126 [21], at 10 µM completely abolished ethanol-induced nuclear Nrf2 expression, which was accompanied by decreased expression of CYP2A6 at both mRNA and protein levels (Fig. 5A, B and C). However, c-Jun N-terminal kinase (JNK) inhibitor, SB600125, showed no effect on ethanol-induced nuclear Nrf2 expression and CYP2A6 induction (Fig. 5D).

## Discussion

**Figure 6:**
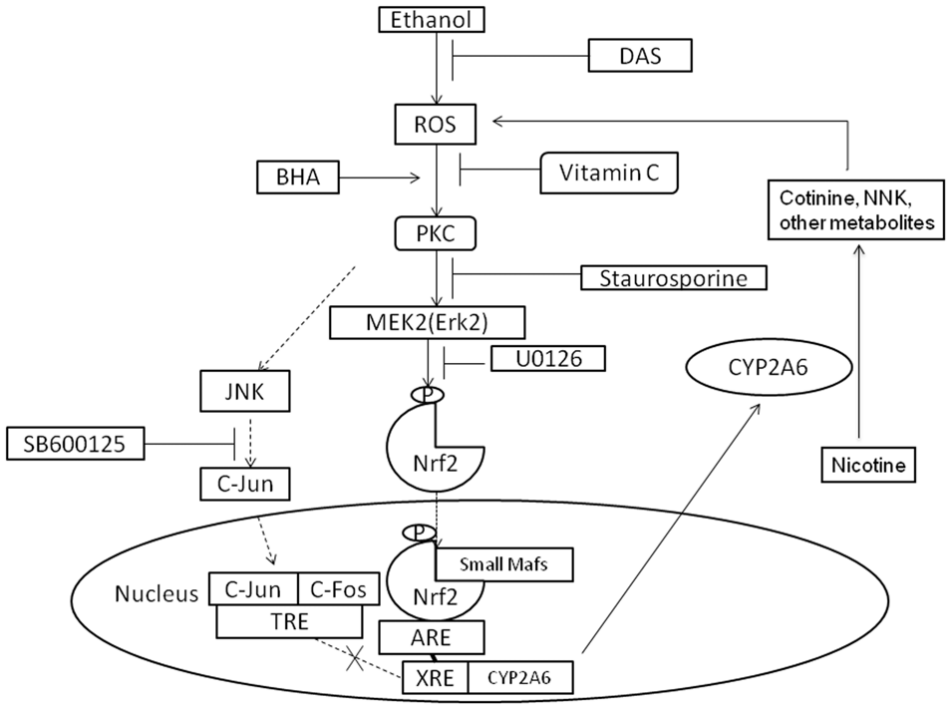
Schematic representation of oxidative stress-dependent Nrf2 translocation through PKC/MEK pathway leading to induction of CYP2A6 by CYP2E1-mediated ethanol metabolism; the CYP2A6 induction can metabolize nicotine and further increase ROS formation. DAS: Diallyl sulfide; ROS: Reactive oxygen species; BHA: Butylated hydroxyanisole; MAPK: Mitogen-activated protein kinase; PKC: Protein kinase C; MEK: Mitogen-activated kinase kinase, Nrf2: Nuclear factor-erythroid2-related factor 2; Erk1: Extracellular signal-regulated kinases; JNK: c-Jun N-terminal kinase; ARE: Antioxidant response element; XRE: Xenobiotic response element; TRE: 12-O-tetradecanoylphorbol-13-acetate response element.

Although CYP2A6 metabolizes the clearance of several xenobiotics, including nicotine, its induction by xenobiotics is not well understood [3], [5], [6]. Recently we have shown that CYP2A6 is induced by ethanol and it metabolizes nicotine into cotinine and NNK, and this produces oxidative stress in U937 cells [12], [13]. In the current study, we show that CYP2A6 is induced by increased oxidative stress mediated through ethanol metabolism by CYP2E1 in U937 monocytes. Furthermore, we have shown that the Nrf2 pathway, which is regulated by PKC and MEK, is involved in CYP2A6 induction mediated by oxidative stress (Fig. 6). Although the involvement of Nrf2 in CYP2A6 induction in hepatic cells has recently been shown [22], this is the first report to provide evidence that ethanol induces CYP2A6 through an oxidative stress-mediated pathway that involves signaling through PKC/MEK/Nrf2.

Although the increase in CYP2A6 expression resulting from a single acute treatment of ethanol is 150–200%, it is significant and these levels are consistent with previous reports using hepatocytes [11], [23], and U937 cells [13]. In this study we used acute ethanol treatment to dissect the mechanism involved in ethanol-mediated regulation CYP2A6 expression. However, it is anticipated that chronic ethanol exposure would produce a relatively greater increase in CYP2E1 expression that would lead to higher levels of oxidative stress and CYP2A6 expression. Indeed, our preliminary observations from human monocytes of mild-to-moderate alcoholics have shown that CYP2E1 and CYP2A6 are induced by ten and four fold, respectively (data not shown), which is consistent with a previous study that evaluated chronic exposure of liver and brain to alcohol and nicotine in rat and monkey [24], [25].

Recently it has been shown that rat CYP2A5 (homologous to human CYP2A6) is induced by increased ROS generated from ethanol metabolism, indicating the central role of oxidative stress in regulating CYP2A5 [23]. A similar mechanism for CYP2A5 induction was also proposed for alcohol-mediated induction of oxidative stress [11]. In these studies, they demonstrated that CYP2A5 induction by ethanol is dependent upon CYP2E1 which metabolizes ethanol and produces ROS. In an independent study with African green monkeys, pretreatment with ethanol elevated nicotine toxicity, presumably through enhanced CYP2A6 expression and nicotine metabolism [25]. These findings are consistent with our results that ethanol-induced oxidative stress by CYP2E1 is responsible for the induction of CYP2A6. The increased level of CYP2A6 could further increase ROS and generate pre-carcinogens through CYP2A6-mediated nicotine metabolism and/or activation of other tobacco constituents as has been demonstrated in liver [26] and U937 monocytes [12].

Oxidative stress generated by BHA has been shown to induce nuclear transcription factor Nrf2, leading to the induction of antioxidants [27], [28]. Consistent with a recent report [22], our results demonstrate that CYP2A6 is induced by ethanol-mediated oxidative stress that subsequently activates the Nrf2 pathway (Fig. 6). An increase in ROS level in the cytoplasm releases Nrf2 from the Nrf2/Kelch-like ECH-associated protein 1(Keap1) complex and the free Nrf2 is phosphorylated by PKC resulting in translocation of Nrf2 from the cytoplasm to the nucleus [29], [30]. The translocated Nrf2 binds antioxidant response elements (ARE) and induces multiple antioxidant enzymes, such as phase II enzymes, NAD(P)H quinone oxidoreductase 1, and glutathione S-transferase (GST) [31], [32]. Earlier studies have shown that the nuclear translocation of Nrf2 and generation of antioxidants are increased by alcohol, and function to ameliorate alcohol-induced apoptotic death and liver toxicity [33], [34]. Furthermore, the Keap1/Nrf2/ARE signaling pathway has been shown to be involved in suppression of nuclear factor kappa-light-chain-enhancer of activated B cells (NF-κB) and its-mediated inflammatory effects [35]. Thus, the protective effect of Keap1/Nrf2/ARE signaling contributes to the maintenance of cellular homeostasis and the prevention of cell and tissue damage. As ARE is a known ligand of AhR [36], Nrf2-mediated CYP2A6 expression may also be involved in the crosstalk between the receptors AhR and PXR.

Staurosporine, which blocks the translocation of Nrf2 into the nucleus, is known to bind PKC leading to the inhibition of phosphorylation of MEK and JNK proteins [37]. Our results from experiments with staurosporine or U0126 (MEK inhibitor) clearly suggest that phosphorylation of MEK, but not JNK, regulates Nrf2-mediated transcription of CYP2A6. It should be noted that U0126 alone also increased the levels of CYP2A6 mRNA, but not the levels of protein. In an earlier study it was shown that, in addition to being a strong inhibitor of MEK, U0126 is also a weak trans-activator of AhR, which binds to the xenobiotic response element (XRE) and upregulates CYP1A enzymes [21]. Since the XRE is common to the CYP1A and CYP2A6 enhancer/promoter regions, U0126 may also induce and/or stabilize CYP2A6 mRNA in the absence of ethanol. However, in the presence of ethanol, U0126 only acts as an inhibitor of MEK because U0126 is a strong inhibitor of MEK, but a weak agonist of AhR.

The findings from our study suggest that ethanol-induced Nrf2 translocation into the nucleus that results into CYP2A6 induction is mediated through the activation of PKC/MEK pathway. PKC is known as a “stress sensor” and controls the functions of other proteins *via* phosphorylation of MEK or JNK. Its activation is involved in multiple signal transduction cascades and is associated with the regulation of different cellular processes; such as, cell growth, immune response, apoptosis, and necrosis [38]. PKC is also known to be involved in alcohol-induced toxicity and liver damage [39]. As the downstream signaling cascades of PKC, both MEK and JNK, have been found to be activated by alcohol leading to expression of antioxidant genes in HepG2 cells and primary hepatocytes [40], [41]. In contrast, other studies have shown that PKC can also directly affect Nrf2 nuclear translocation and regulate the expression of antioxidant genes in HepG2 cells [42]–[44]. These contrasting findings suggest that the involvement of MEK in PKC-mediated Nrf2 translocation is cell type dependent.

The finding of oxidative stress-mediated CYP2A6 expression by ethanol in monocytes, which are known to secrete anti-inflammatory cytokines and chemokines [45], is important because of the potential for cross talk between CYP and cytokines. In fact, ethanol has been shown to induce several cytokines in monocytes, lymphocytes, and astrocytes [46]. Furthermore, emerging studies from simultaneous expressions of CYPs and cytokines [47], as well as the finding that IL-6 regulates CYP3A4, suggest a cross talk between CYPs and cytokines [48]. This could have important implications in the case of simultaneous exposure to various xenobiotics, as well as bacterial or viral pathogens. Therefore, it is imperative to further dissect the signaling pathway that is responsible for the simultaneous induction of both CYP2A6 and cytokines by ethanol in monocytes.

Previously, lipid peroxidation has been observed in studies on the effects of alcohol [49], [50]. In addition, nicotine has been reported to cause oxidative damage and to increase the permeability of the blood-brain barrier [51]. Chronic accumulation of ROS leads to over consumption of glutathione and antioxidants, leading to cellular toxicity [52]. The use of antioxidant supplements, such as vitamin C and E, has been shown to be effective in attenuating the oxidative stress-mediated effects of alcoholic liver disease [53]. Our findings suggest the potential use of a CYP2E1 selective inhibitor [19] among alcohol and tobacco users to reduce alcohol-mediated oxidative stress and induction of CYP2A6 expression and activity, which can further increase oxidative stress through nicotine metabolism. Our argument is supported by the fact that a similar approach using a selective CYP2A6 inhibitor is being considered to treat nicotine dependence [54] and to prevent tobacco/nicotine-mediated lung cancer [55].

In conclusion, the current study provides the first report of the regulation of CYP2A6 expression by ethanol through activation of the oxidative stress-mediated PKC/MEK/Nrf2 pathway in monocytic cells (Fig. 6). This is a significant finding because tobacco/nicotine has been shown to enhance HIV-1 replication in alveolar macrophages and microglia [56], [57], perhaps through oxidative stress [58]–[60]. We speculate that ethanol-mediated induction of CYP2A6 will increase the metabolism of nicotine leading to an increase in oxidative stress and perhaps HIV-1 replication in HIV-infected individuals who consume both alcohol and tobacco (Fig. 6). This line of investigation may have clinical relevance because HIV-1 infected monocytes may infiltrate the CNS and exacerbate neuroAIDS and damage neurons [61]. Further, since the prevalence of smoking is 3 times higher among alcoholics, especially among HIV-infected individuals, this finding has important implications for HIV+ alcohol/tobacco co-abusers.
